# Cone Beam Computed Tomography-aided Endodontic Management of Second Maxillary Molar with Two Separate Palatal Roots: A Case Report

**DOI:** 10.7759/cureus.7347

**Published:** 2020-03-20

**Authors:** Purushotham M, Hrudi Sahoo

**Affiliations:** 1 Conservative Dentistry and Endodontics, Sathyabama Dental College and Hospital, Chennai, IND

**Keywords:** cone beam computed tomography, endodontics, root canal treatment, maxillary molars, palatal roots, extra palatal root, wave one gold, reciprocating files, case report, mesial palatal root

## Abstract

An infected human root canal system harbors harmful microbiota that needs to be eliminated by root canal therapy. But the human root canal system is known to have a complex anatomy. Hence, the knowledge of a possibly aberrant anatomy is deemed necessary before carrying out a root canal therapy. Usually, the maxillary second molars have three roots and three or four root canals (a second mesiobuccal canal as the fourth canal). The presence of a second palatal root and a second palatal root canal is very rare (1.4% incidence). Failure to locate and treat such aberrant external and internal anatomy results in a compromised root canal therapy. With the introduction of advanced imaging and visualization techniques such as cone beam computed tomography (CBCT) and dental operating microscopes, the variations in root anatomy have been successfully detected and treated. This case report describes the endodontic management of a second maxillary molar with two palatal roots and four separate canals, performed with the help of CBCT.

## Introduction

Endodontic therapy is performed to eliminate viable bacteria and toxins from the human root canal system. The challenging anatomy of the root canal complicates this task. For this reason, a thorough knowledge of the aberrant root canal anatomy is highly important. Failure to locate and treat such variations will lead to incomplete instrumentation, inadequate cleaning, insufficient canal obturation, and the presence of untreated canals [1]. Such untreated canals harbor harmful microorganisms that lead to failure of the root canal therapy.

Upon review of the literature, it is determined that the second maxillary molar anatomically resembles the first maxillary molars, with the only difference being in root orientation. The roots in a maxillary second molar are either fused or united closer, unlike the first maxillary molar. Very often, the roots are also curved to a greater extent when compared to their first molar counterpart. The first and second maxillary molars have three roots, namely, mesial, buccal, and palatal. Both the molars have one canal in each root with mesial root as an exception. In the first maxillary molars, the possibility of finding a second mesiobuccal canal in a single mesiobuccal root can range from 18-96% [2].

In the second maxillary molar, unlike the first molar, an imaginary flat triangle is formed when all the three canal orifices are joined and, very often, a straight line can also be seen. The canal orifices are slightly funnel-shaped and the floor of the pulp chamber is convex [3]. A review of the literature suggests that very few case reports exist on anatomic variations in the maxillary second molar [4-7]. The frequency of a second palatal root in the maxillary second molar can be as low as 1.4% [8]. This article presents a case of a maxillary second molar with a second separate palatal root canal orifice and a second palatal root.

## Case presentation

A 33-year-old male reported to our Conservative Dentistry and Endodontics department with the chief complaint of severe pain in the upper left back teeth region for the past three days. The pain was exaggerated on eating hot or cold food items. His past dental history revealed that a root canal therapy had been initiated by a private practitioner a week ago. On clinical examination, #26 was missing and an old restoration was seen in relation to #27. According to the patient’s complaint and history, an incomplete root canal therapy in relation to #26 was decided to be the final diagnosis.

**Figure 1:**
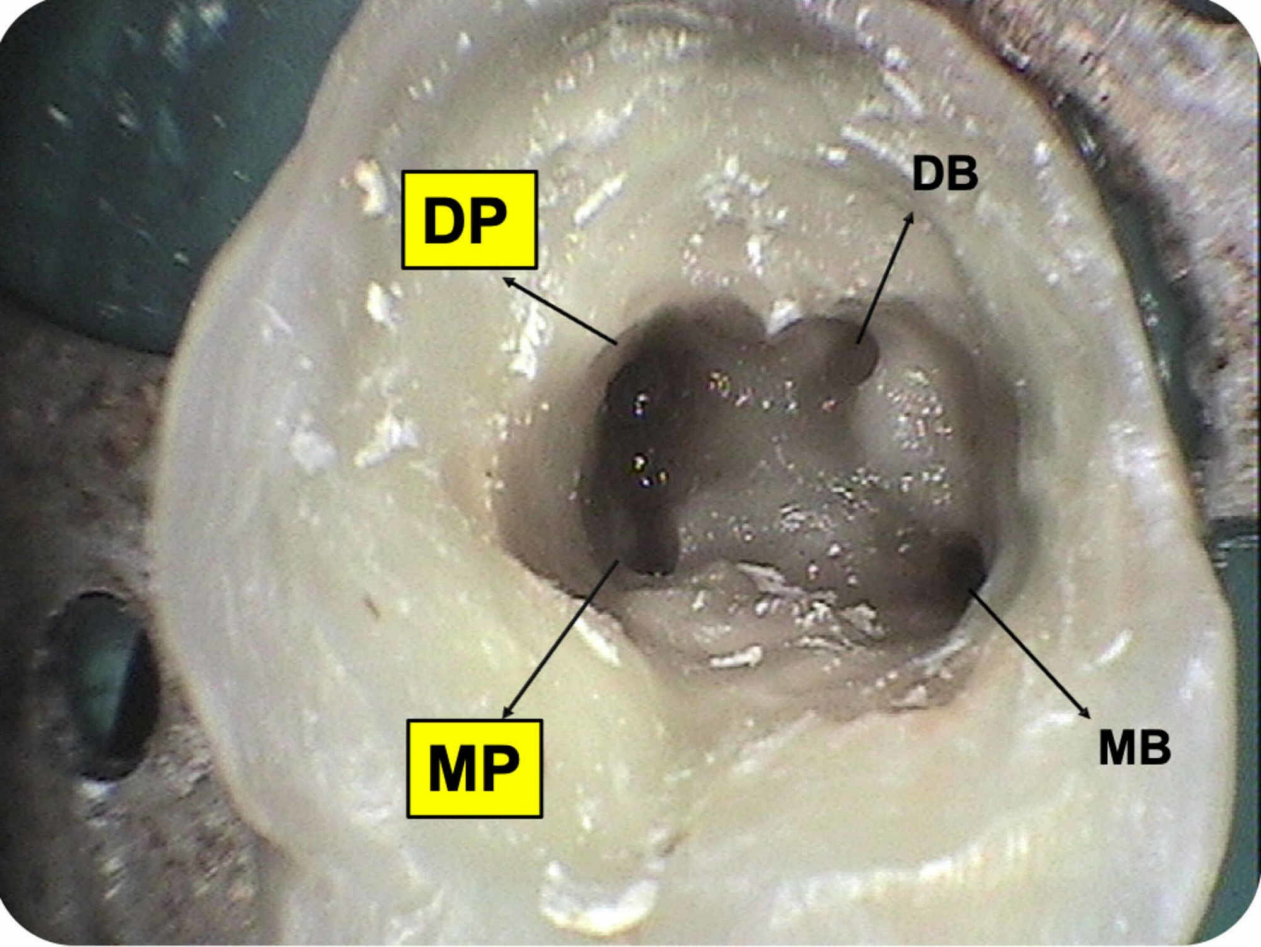
Access cavity of maxillary second molar (#27) DP: distopalatal canal orifice; MP: mesiopalatal canal orifice; DB: distobuccal canal orifice; MB: mesiobuccal canal orifice

**Figure 2:**
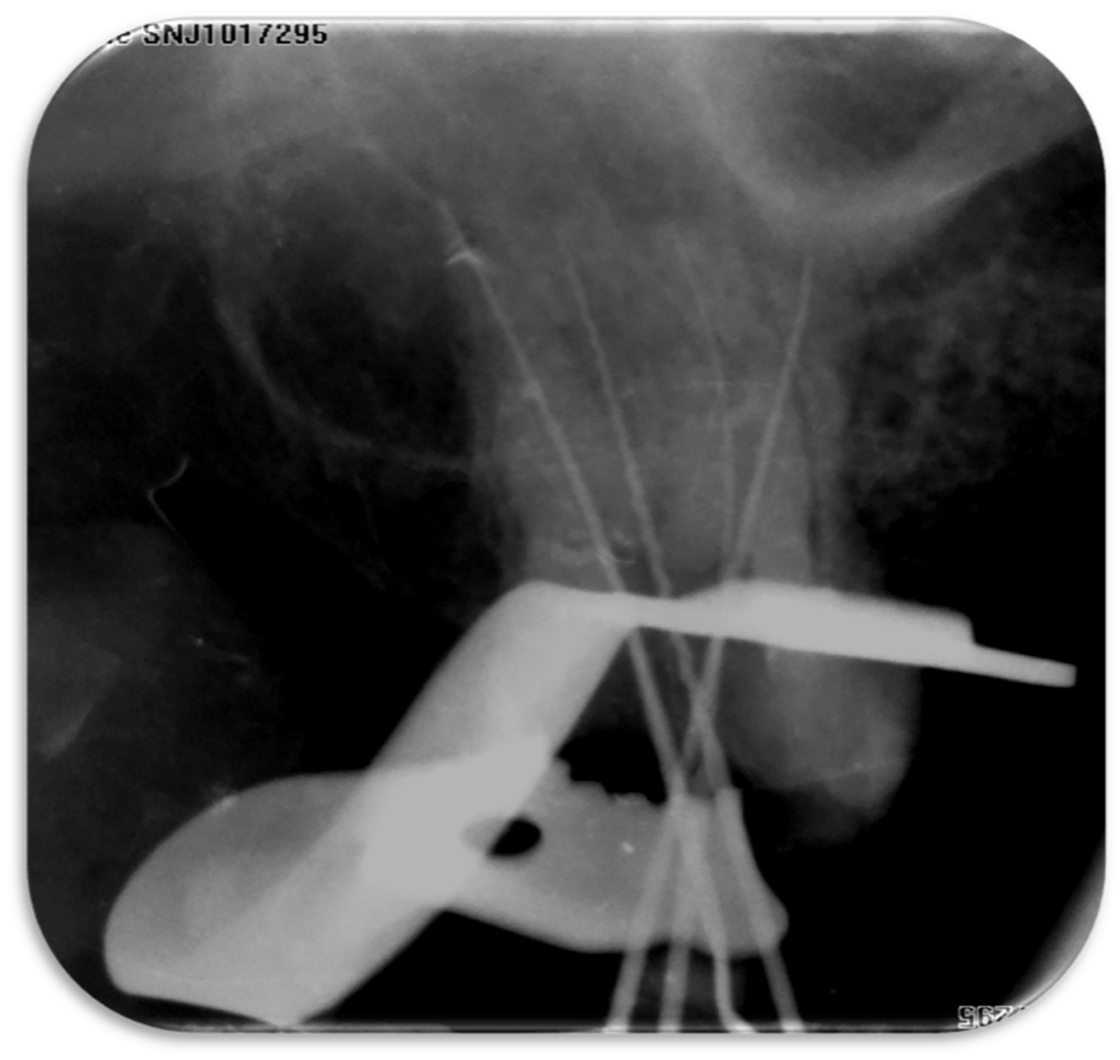
Radiographic working length determination (#27)

After obtaining informed consent from the patient, the old coronal restoration was removed and the access cavity was irrigated with normal saline. Bleeding was evident in the pulpal floor, suggestive of incomplete pulp extirpation or a possible presence of an extra-canal. On careful examination of the access cavity, three root canal orifices were located, i.e mesiobuccal, distobuccal, and palatal root canals. Under a dental operating microscope with a 5X magnification (Seiler Medical, St. Louis, MO), the floor of the access cavity was examined using a DG-16 explorer (Hu-Friedy, Chicago, IL). Although the second mesiobuccal canal was not located, it was searched for by troughing along the line joining mesiobuccal and palatal orifices with low-speed burs and ultrasonic tips. The palatal canal unusually seemed to be distally positioned and a bleeding point was noticed mesial to the palatal canal orifice (Figure 1). A hand k-file (MANI, Inc., Shioya, Japan) (#10, 25 mm) was placed inside and an apex locator was used to confirm the presence of an extra palatal canal. A working length radiograph was taken (Figure 2). Pulp was completely extirpated and the access cavity was temporized with Cavit (3M Espe AG, Seefeld, Germany). To rule out the presence of further complexities inside the extra palatal canal, following the “as low as reasonably achievable” (ALARA) principles, a low-field-volume cone beam computed tomography (CBCT) scan was advised.

**Figure 3:**
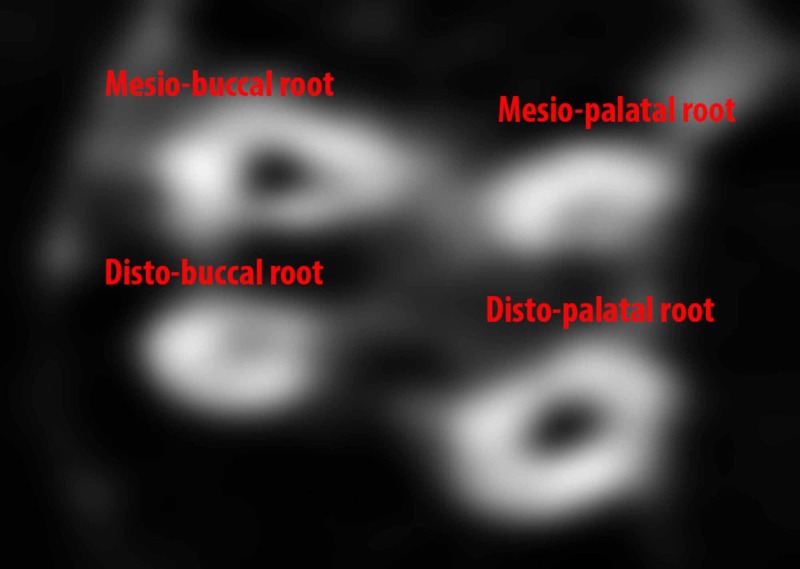
CBCT axial image of #27 CBCT: cone beam computed tomography

**Figure 4:**
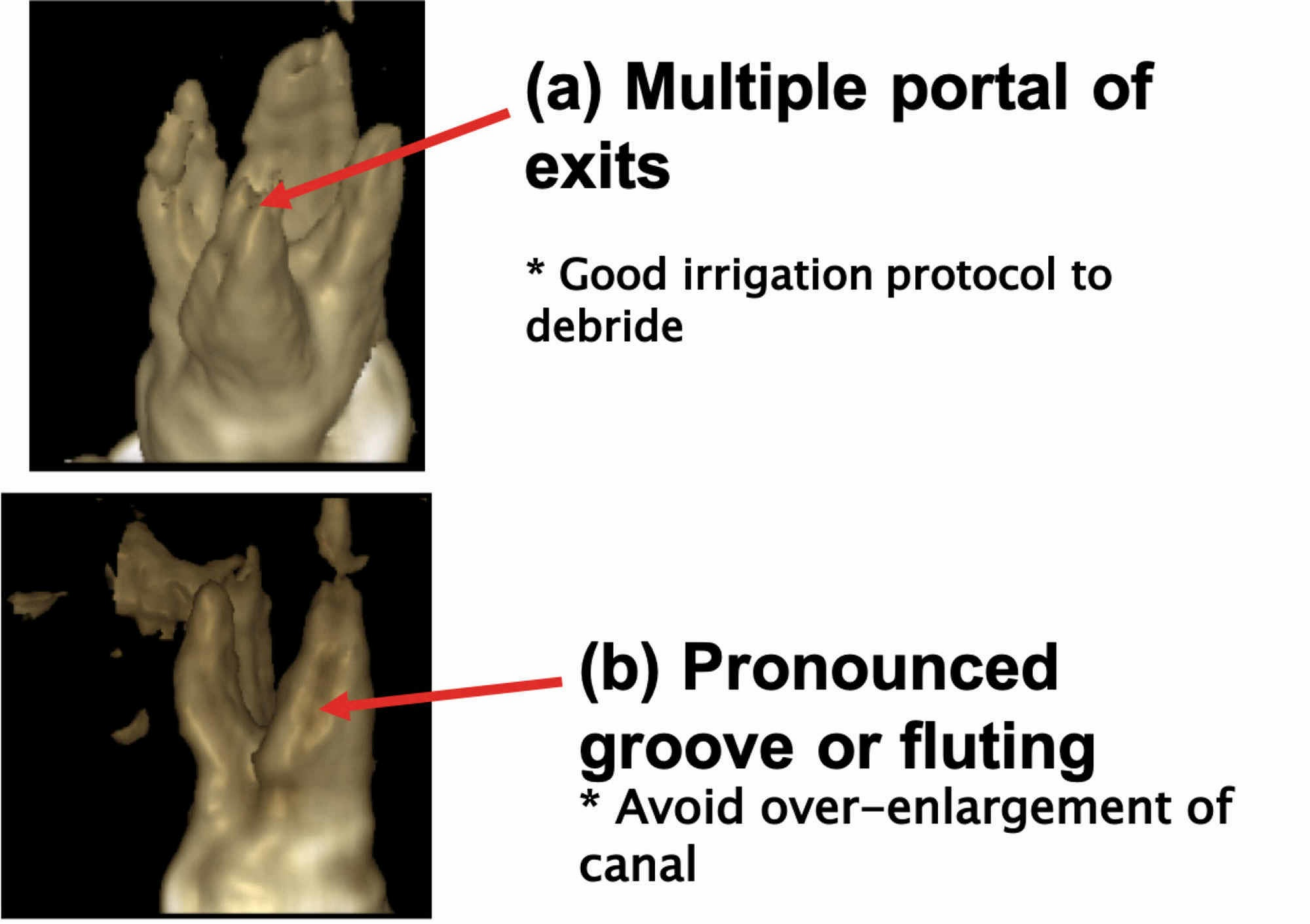
3D reconstruction of #27 CBCT images CBCT: cone beam computed tomography

In the subsequent visit after a week, the CBCT axial images were evaluated. The CBCT image revealed an extra palatal root mesial to the large palatal root and was, hence, termed as mesiopalatal root (Figure 3). The distopalatal root canal orifice was large and oval in shape when compared to the mesiopalatal root canal orifice. The distopalatal root canal had a single exit apically. On a 3D reconstruction of the CBCT images, the mesiopalatal root showed numerous accessory exits towards the apical third, and a pronounced grooving or fluting was seen on the outer surface of the mesiopalatal root (Figure 4). In light of the above findings, all the root canals were chemically debrided using saline and sodium hypochlorite. The root canals were mechanically debrided and shaped using WaveOne Gold (Dentsply Sirona, York, PA) files.

**Figure 5:**
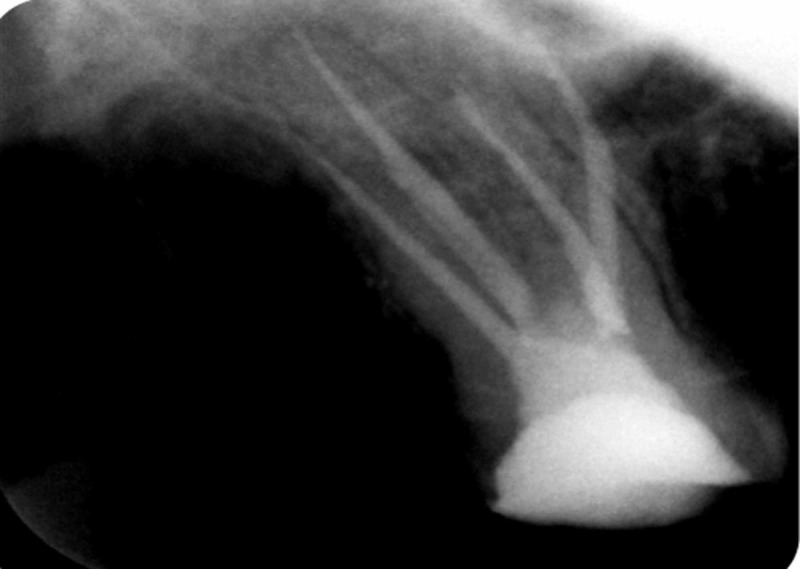
Obturation radiograph of #27

**Figure 6:**
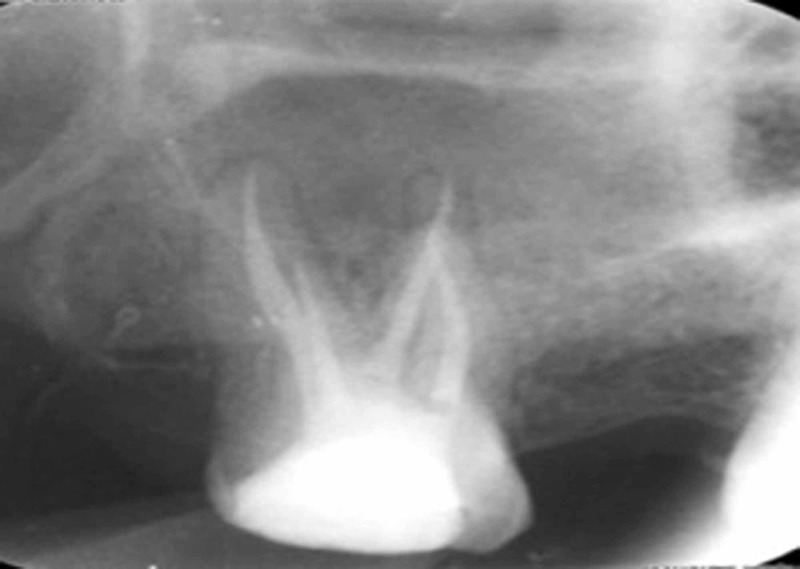
Follow-up radiograph of #27 after six months

After a period of seven days, the patient was asymptomatic and the root canals were completely obturated using thermo-plasticized gutta-percha (Elements; VDW, Munich, Germany), and a postoperative intra-oral radiograph was taken (Figure 5). A six-month follow-up radiograph was also taken, which revealed a possible successful outcome of the treatment (Figure 6).

## Discussion

Together with the diagnosis and treatment planning, a thorough knowledge of the root canal system and its frequent variations is necessary for successful root canal treatment. Stone and Stroner have reported two variations in the palatal root of maxillary molar [9]. Firstly, the palatal root could exhibit two canals with separated or bifurcated canals in a single root and, secondly, two canals in two separated roots. In another classification by Christie et al., the palatal roots were divided into three types: type 1 - cow-horn-shaped two buccal roots and two long, tortuous, widely divergent palatal roots; type 2 - four roots that are separate, short, and parallel to each other with blunt apices separated roots of blunted root apices that run parallel; type 3 - root morphology is constricted with mesiobuccal, mesiopalatal, and distopalatal canals engaged in one web of root dentin. The distobuccal canal root seems to stand alone and may diverge to distobuccal. The present case had four separate roots (two buccal and two palatal) with four canals which belonged to the type I category of Christie et al. classification [10].

Missed and untreated root canals inevitably lead to failure of the treatment. Hence, it is always necessary to judiciously use the latest technology advancements like CBCT and dental microscopy. According to the 2012 American Association of Endodontists (AAE) position statement on the use of microscopes and other magnification techniques, dental microscopes play a major role in locating missed canals. Baratto Filho et al. have proposed that operating microscope and CBCT can be important for locating and identifying root canals and that CBCT can be used as a good method for the initial identification of maxillary first molar internal morphology [4].

Further, to study the morphology of the mesiopalatal canal, the CBCT images were reconstructed using Horos Dicom visualization software. The images revealed multiple portals of exit at the apical third of the mesiopalatal canal and also a pronounced grooving or fluting on the mesial surface of the mesiopalatal root. These morphologic variations need to be kept in mind while preparing the root canal as excess root dentin removal during shaping procedures may be detrimental, and advanced irrigation devices such as EndoVac (Kerr, Brea, CA) should be used for improved debridement of such complex canal anatomy.

## Conclusions

Unusual root morphologies and canal configurations occur very rarely and the clinician should be aware of the same. A meticulous treatment plan, including careful interpretation of different angles of preoperative radiographs, following and implementing the principles of access cavity preparation, and the use of available latest technology like CBCT and microscopes, is very important in treating aberrant anatomies in the root canal with an improved prognosis.

## References

[1] Siqueira JF Jr, Rôças IN (2008). Clinical implications and microbiology of bacterial persistence after treatment procedures. J Endod.

[2] Cleghorn BM, Christie WH, Dong C (2006). Root and root canal morphology of the human permanent maxillary first molar: a literature review. J Endod.

[3] Hargreaves K, Cohen S (2010). Cohen’s Pathways of the PULP.

[4] Baratto-Filho F, Fariniuk LF, Ferreira EL, Pecora JD, Cruz-Filho AM, Sousa-Neto MD (2002). Clinical and macroscopic study of maxillary molars with two palatal roots. Int Endod J.

[5] Barbizam JV, Ribeiro RG, Tanomaru Filho M (2004). Unusual anatomy of permanent maxillary molars. J Endod.

[6] Benenati FW (1985). Maxillary second molar with two palatal canals and a palatogingival groove. J Endod.

[7] Alani AH (2003). Endodontic treatment of bilaterally occurring 4-rooted maxillary second molars: case report. J Can Dent Assoc.

[8] Peikoff MD, Christie WH, Fogel HM (1996). The maxillary second molar: variations in the number of roots and canals. Int Endod J.

[9] Stone LH, Stroner WF (1981). Maxillary molars demonstrating more than one palatal root canal. Oral Surg Oral Med Oral Pathol.

[10] Christie WH, Peikoff MD, Fogel HM (1991). Maxillary molars with two palatal roots: a retrospective clinical study. J Endod.

